# Real-Time Vibration Energy Prediction for Semi-Active Suspensions Using Inertial Sensors: A Physics-Guided Deep Learning Approach

**DOI:** 10.3390/s26051695

**Published:** 2026-03-07

**Authors:** Jian Cheng, Fanhua Qin, Leyao Wang, Ruijuan Chi

**Affiliations:** 1 School of Mechanical and Electrical Engineering, North China Institute of Aerospace Engineering, Langfang 065000, China; szy66@nciae.edu.cn (J.C.); m18910760691@163.com (F.Q.); 13811453263@163.com (L.W.); 2 College of Engineering, China Agricultural University, Beijing 100083, China

**Keywords:** semi-active suspension, inertial sensors, physics-guided deep learning, vibration energy prediction, continuous wavelet transform, embedded AI

## Abstract

**Highlights:**

**What are the main findings?**

- A Physics-Informed Gated CNN (PI-GCNN) framework is proposed that integrates continuous wavelet transform (CWT) features with an asymmetric sparse physics loss to accurately predict multi-modal vibration energy.
- Experimental validation on the PVS 9 real-vehicle dataset demonstrates that the model achieves a significant predictive phase lead of 100–200 ms and a low inference latency of 0.20 ms.

**What are the implications of the main findings?**

- The achieved phase lead creates a critical actuation window for semi-active suspensions, enabling a shift from reactive feedback to proactive feedforward control to effectively mitigate road impact shocks.
- With a compact parameter size of 0.10 M, the proposed algorithm offers a computationally efficient solution feasible for real-time deployment on resource-constrained automotive embedded chips.

**Abstract:**

Response latency and sensor noise are universal challenges in closed-loop control systems. In the context of semi-active suspensions, these issues also exist and manifest as critical bottlenecks. Due to the highly transient nature of road shocks, the inherent physical actuation delays of the hardware, combined with the phase lag introduced by traditional signal filtering, often cause the control response to significantly lag behind the physical excitation. To address this issue from a predictive perspective, this study proposes a Physics-Informed Gated Convolutional Neural Network (PI-GCNN) designed to predict future multi-modal energy evolution, thereby enabling feedforward control. Unlike traditional feedback mechanisms, the proposed framework employs the Continuous Wavelet Transform (CWT) to convert short-horizon inertial data into time–frequency scalograms, effectively isolating transient shock features from background vibrations. A novel physics-guided gating mechanism is embedded within the network architecture to regulate feature activation. This mechanism is trained using an asymmetric sparse physics loss, which combines L1 regularization with adaptive spectral consistency constraints to enforce noise suppression on flat roads while ensuring sensitivity to impacts. Extensive validation was conducted using high-fidelity heavy truck simulations and the public PVS 9 real-world dataset. The results confirm that the PI-GCNN achieves a predictive phase lead of approximately 100–200 ms over real-time baselines, creating a valuable actuation window for suspension dampers. Furthermore, the model demonstrates exceptional computational efficiency, with a parameter count of 0.10 M and a single-frame inference latency of 0.25 ms, making it highly suitable for deployment on resource-constrained automotive edge computing platforms.

## 1. Introduction

The vibration isolation performance of vehicle suspension systems is critical for ensuring ride comfort and handling stability. In modern automotive engineering, the primary challenge lies in resolving the inherent conflict between isolating high-frequency road irregularities and maintaining low-frequency tire-road contact. As demonstrated by Knap et al. [1] in their recent study on hydraulic dampers with piezoelectric valves, the efficacy of vibration reduction relies heavily on the responsiveness of the actuation mechanism. Similarly, Xie et al. [2] explored low-frequency active vibration isolation for precision payloads, emphasizing the necessity of independent modal control to manage complex vibration environments. While active and semi-active suspensions offer theoretical advantages over passive systems, their practical performance is often constrained by the control strategy. Huang et al. [3] highlighted this in their design of a suspension leveling system for agricultural machinery, where simulation results underscored the importance of adaptive control algorithms. To address these control challenges, various intelligent approaches have been proposed. Khan et al. [4] introduced a neuro-fuzzy wavelet network for full-car active suspension, while Zhang et al. [5] developed a fuzzy wavelet neural network controller optimized by genetic algorithms. Despite these advancements, Xie et al. [6] pointed out in their dynamic load analysis of multi-axle vehicles that traditional feedback control inherently suffers from phase lag. This latency occurs because the controller reacts only after the vibration has been transmitted through the tires to the vehicle body sensors, rendering the system passive to sudden impulsive shocks.

To mitigate the limitations of feedback latency, predictive control strategies that utilize “look-ahead” information have garnered significant attention. These methods typically rely on external environmental perception. Hegde et al. [7] proposed an ensemble learning approach for detecting road damage, and Fortin et al. [8] utilized UAV-assisted terrain awareness for off-road navigation. However, visual or LiDAR-based solutions are susceptible to lighting and weather conditions. Consequently, vibration-based sensing methods have emerged as a robust alternative. Cui et al. [9] recently developed a road surface recognition algorithm based on vehicle vibration data using a 1D-CNN, demonstrating high accuracy in classifying road types. Furthermore, Chang et al. [10] investigated the use of MEMS acceleration sensors embedded in pavement for vehicle detection, validating the feasibility of vibration-based monitoring. Similarly, Mehta et al. [11] presented a bicycle sensor platform combining physics-based and machine-learning approaches for anomaly detection. Nevertheless, processing these sensor signals presents its own set of challenges. Haghighi et al. [12] noted the difficulties in noise source identification using purely data-driven methods, while Karballaeezadeh et al. [13] discussed the potential of AI in modeling road roughness but cautioned against the complexity of real-world vibration data.

A critical issue in vibration-based prediction is the extraction of effective features from noisy sensor data. Time-domain analysis often fails to detect the onset of a shock signal until it exceeds the noise floor. To overcome this, frequency and time–frequency analysis (TFA) techniques have been widely adopted. Huber et al. [14] showed that frequency analysis is efficient for detecting automotive damper defects. To further enhance feature resolution, Yin et al. [15] proposed a velocity-guided Chirplet transform. The success of spectrogram-based feature extraction has also been proven in other domains; for instance, Luitel and Liu [16] applied spectrogram representations to audio sentiment analysis, and Kumar G et al. [17] developed a multi-resolution channel attention transformer for acoustic detection. In the field of mechanical prognostics, Yoo and Baek [18] utilized continuous wavelet transform (CWT) for bearing lifetime prediction, and Rajabioun and Atan [19] optimized source selection for bearing fault classification. Gao [20] also integrated wavelet transform with digital twins for surface roughness prediction. Moreover, advanced deep learning tools for TFA have been developed, such as WVDNet by Liu et al. [21], TFA-Net by Pan et al. [22], and the high-quality analysis method by Zhao et al. [23], all of which facilitate the extraction of deep features from complex non-stationary signals. Battulga et al. [24] further validated the use of vibration-based detection for mechanical defects in motor systems.

Although Deep Neural Networks (DNNs) excel at feature extraction, they often lack generalization capability when training data is limited or when facing unseen operating conditions. To address this, the Physics-Informed Neural Network (PINN) framework, originally introduced by Raissi et al. [25], integrates physical laws into the learning process. This paradigm has shown remarkable versatility in recent years. Xu et al. [26] applied PINNs to vibration-aware trajectory optimization for mobile robots, and Lao et al. [27] utilized them for vibration control of piezoelectric cantilever beams. In terms of system stability, Kokolakis et al. [28] proposed a safe physics-informed learning approach for predefined-time stabilization. The fusion of data and physics has also proven effective in prognostics and diagnostics; Jiang et al. [29] developed a knowledge-data collaborative model for gear degradation, while Chen et al. [30] proposed an explainable deep ensemble model for bearing faults. Liu and Huang [31] employed PINNs for underwater vehicle tracking, and Huai et al. [32] developed a PINN-based observer for intermittent fault detection. Furthermore, Liu et al. [33] applied a neural network optimized by genetic algorithms for alignment prediction in marine vibration isolation systems, and Majumder et al. [34] demonstrated the efficiency of PINNs in obstacle avoidance for autonomous vehicles. These studies collectively indicate that embedding physical constraints into neural networks can significantly improve model robustness and physical consistency.

In this context, this paper proposes a Physics-Informed Gated Convolutional Neural Network (PI-GCNN) for suspension predictive control. Utilizing the PVS 9 passive vehicular sensor dataset provided by Menegazzo and von Wangenheim [35], this study aims to predict future shock events by analyzing the time–frequency texture of current vibration signals. Unlike traditional methods, our approach embeds physical priors—specifically signal sparsity and energy consistency—into the network architecture via a custom gating mechanism and an asymmetric loss function. This design ensures the model remains sensitive to impending shocks while minimizing false triggering on flat roads, thereby solving the phase lag problem inherent in feedback systems. Functioning strictly as an upstream perception module, the predicted energy matrix provides a universal look-ahead reference that can be integrated with various semi-active control laws (e.g., Sky-hook) to proactively compensate for the mechanical actuation delays of physical hardware (e.g., MR dampers).

The remainder of this paper is organized as follows. Section 2 details the proposed methodology, specifically focusing on the PI-GCNN architecture, the physics-guided gating mechanism, and the design of the asymmetric sparse physics loss. Section 3 describes the high-fidelity simulation setup and presents a comparative analysis of the model’s performance against baselines under various road conditions. Section 4 provides experimental validation using real-world vehicle datasets and evaluates the computational efficiency for embedded deployment. Finally, Section 5 summarizes the main findings and outlines future research directions.

## 2. Methodology

**Figure 1:**
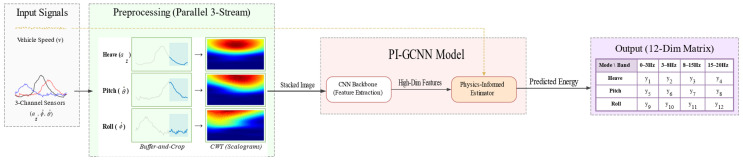
The overall framework of the proposed multi-modal energy prediction system.

This paper presents a Physics-Informed Gated Convolutional Neural Network (PI-GCNN) framework designed to address the issues of response latency and false triggering inherent in semi-active suspension control. By establishing a physically constrained mapping mechanism from time–frequency observations to multi-band energy, the framework extracts transient spectral features from short-horizon sensor sequences to predict the future energy distribution of the vehicle across different dynamic modes. The proposed methodology comprises three key elements: high-fidelity time–frequency preprocessing utilizing a buffer-based Continuous Wavelet Transform (CWT), a speed-aware physics-gated network that integrates kinematic state feature extraction with a gating structure, and an asymmetric physics-guided loss function designed to guide model optimization. The runtime data flow of the system is illustrated in Figure 1, which demonstrates the end-to-end inference process from raw sensor signal input, via parallel time–frequency transformation, to the final mapping of the multi-modal energy matrix.

### 2.1. Problem Formulation: Multi-Modal Matrix Prediction

The vibration response of a vehicle in motion is a multi-dimensional coupled dynamic process. To achieve precise semi-active control, we predict the future energy evolution of three key dynamic modes across four frequency bands. We define the prediction target as a multi-modal spectral energy matrix $Y\in R^{3\times 4}$:

(1) $$Y=\left[\begin{array}{cccc} y_{heave,0-3} & y_{heave,3-8} & y_{heave,8-15} & y_{heave,15-20}\\ y_{pitch,0-3} & y_{pitch,3-8} & y_{pitch,8-15} & y_{pitch,15-20}\\ y_{roll,0-3} & y_{roll,3-8} & y_{roll,8-15} & y_{roll,15-20} \end{array}\right]$$

where the row vectors correspond to the heave (*Z*), pitch (*θ*), and roll (*φ*) modes, respectively. The column vectors correspond to four frequency bands divided based on vehicle dynamics theory and the ISO 2631-1 standard [36]:

- Primary Ride Frequencies/Sprung Mass Modes (0–3 Hz): Covers the sprung mass rigid body modes of most wheeled vehicles. Although natural frequencies vary among vehicle types (e.g., passenger cars typically range from 1.0 to 1.5 Hz, whereas heavy vans may extend to 1.5–2.5 Hz), this range effectively encapsulates the primary low-frequency energy determining ride comfort and handling stability.
- Human Sensitivity (3–8 Hz): Represents the resonance zone where the human spine and abdominal organs are most sensitive to vertical vibration [36]. Suppressing energy transmission in this band is the core objective of semi-active suspension control.
- Wheel Hop/Unsprung Mass (8–15 Hz): Corresponds to the resonance frequency of the unsprung mass (tires and axles). This band serves as a key window for monitoring tire-ground contact stability and driving safety.
- High-Frequency Jitter (15–20 Hz): Used to capture road texture noise and structural high-frequency vibrations.

Input Horizon Selection: When determining the input history window length $T_{eff}$, a trade-off between information completeness and real-time responsiveness is required. When processing impulsive road shocks, an excessively long window introduces buffering latency and leads to feature dilution. Accordingly, this paper sets the effective input window to $T_{eff}=0.1\;s$.

The input vector is defined as follows:

(2) $$X_{in}=\{S_{obs}\in R^{3\times F\times T_{eff}},v\}$$

where $S_{\mathrm{obs}}$ denotes the tri-axial inertial sensor observation sequence after time–frequency transformation within the past 0.1 s, and $\nu \in R^{1}$ represents the current vehicle speed. The objective is to construct a mapping function $F_{\Theta }$ to predict the energy matrix $\hat{Y}=F_{\Theta }(S_{obs},v)$ within the future $T_{\mathrm{pred}}=0.5\;s$, providing a feedforward compensation signal for the controller.

### 2.2. High-Fidelity Time–Frequency Representation

#### 2.2.1. Continuous Wavelet Transform (CWT)

Given the significant non-stationary and transient nature of road impact signals, this study employs the Continuous Wavelet Transform (CWT) as a front-end processor. The Complex Morlet Wavelet (cmor 1.5–1.0) is selected as the basis function, providing a reasonable balance between time and frequency localization. Its analytic nature further allows for the extraction of a relatively smooth energy envelope for modal analysis:

(3) $$\psi (t)=\frac{1}{\sqrt{\pi f_{b}}}e^{2i\pi f_{c}t}e^{-\frac{t^{2}}{f_{b}}}$$

where $f_{b}=1.5$ and $f_{c}=1.0$. The core advantage of CWT lies in its capacity to capture spectral precursors. Before the time-domain acceleration amplitude peaks, the sudden high-frequency energy changes generated at the instant of wheel-obstacle contact are detected by the CWT, thereby overcoming the inherent lag of physical detection.

#### 2.2.2. Boundary Effect Mitigation Strategy

**Figure 2:**
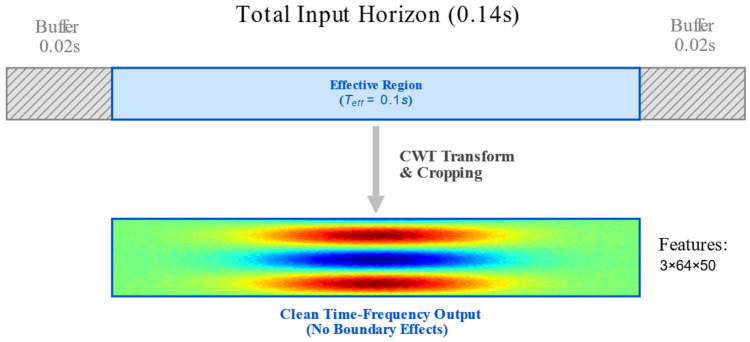
Schematic of the “Buffer-and-Crop” strategy for mitigating the cone of influence effect in CWT.

To address the cone of influence (COI) effect inherent to CWT at the edges of finite-length signals, we implement a “Buffer-and-Crop” strategy, as illustrated in Figure 2.

The specific procedure is as follows: Although model inference requires only $T_{eff}=0.1\;s$ of data, during the sampling phase, we extract a signal segment with a total length of $T_{\mathrm{total}}=0.14\;s$, denoted as $X_{total}\;=\;\left[x_{\text{pad\_left}},x_{eff},x_{\text{pad\_rig}ht}\right]$, incorporating Δt=0.02 s of buffer data on both the left and right margins. Upon completion of the transformation, the edges contaminated by the COI are cropped, retaining only the central 0.1 s high-fidelity region $S_{eff}$. This strategy mathematically ensures the authenticity of the input features.

Prior to being fed into the PI-GCNN, these cropped time–frequency matrices were subjected to a logarithmic transformation (log(1+x)) to compress extreme energy peaks. Subsequently, instance-level Z-score standardization was applied to each channel to zero-center the features and scale them to a unit variance. This procedure ensures a uniform feature scale and accelerates model convergence.

### 2.3. Physics-Informed Gated CNN Architecture

**Figure 3:**
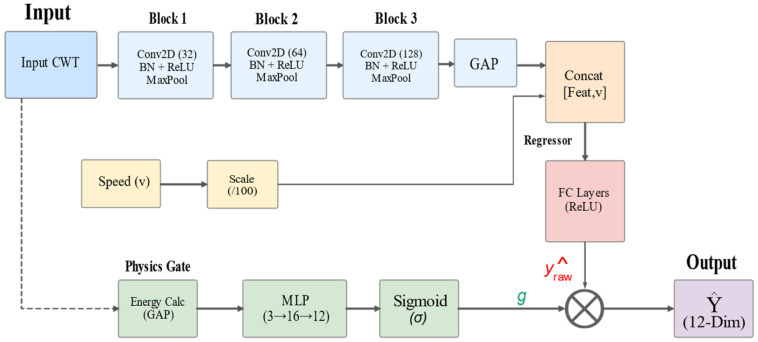
Detailed architecture of the Physics-Informed Gating Mechanism and the computation of Sparse Physics Loss.

To accommodate complex road scenarios, this study designs a Physics-Informed Gated CNN (PI-GCNN). As illustrated in Figure 3, the architecture stacks tri-axial sensor data into a 3-channel tensor $X\in R^{3\times H\times W}$, leveraging 2D convolutions to extract inter-channel coupling features.

The backbone network consists of three stacked convolutional blocks (Conv-Blocks), utilizing standard 3×3 kernels. A Global Average Pooling (GAP) layer is introduced at the end to compress the 2D feature map into a 1D vector. Considering the Doppler effect in vehicle dynamic responses (f∝v), we concatenate the scaled vehicle speed $v^{\prime }$ with the feature vector extracted by the CNN, enabling the regressor to dynamically adjust the attention weights for features in different frequency bands based on velocity. To address false high-energy predictions on flat roads, we design a parallel physics-gating branch:

- Global Energy Perception: Calculates the global average energy ${\bar{E}}_{\mathrm{in}}$ of the input CWT map.
- Hard Threshold Generation: A gating coefficient g∈(0,1) is generated via an MLP. We introduce a strong negative bias at the output layer to ensure the gate defaults to a suppressed state during initialization:

(4) $$g=\sigma (\mathrm{MLP}({\bar{E}}_{\mathrm{in}})-\beta )$$

where β is a learnable or fixed bias parameter.

- Prediction Correction: The final output is defined as $\hat{y}={\hat{y}}_{raw}\cdot g$.

### 2.4. Sparse Physics Loss: An Asymmetric Synergistic Mechanism

To internalize physical priors into model parameters, we construct a loss function comprising triple constraints. Its core design lies in the “asymmetric synergy” between the L1 sparsity term and the adaptive spectral consistency term:

(5) $$L_{\mathrm{total}}=L_{\mathrm{Fidelity}}+\lambda_{\mathrm{phy}}L_{\mathrm{Consistency}}+\lambda_{\mathrm{sparse}}L_{\mathrm{Sparsity}}$$

- Data Fidelity Term

The Huber Loss is adopted to enhance model robustness against extreme shock samples (outliers) and prevent gradient explosion.

- Sparsity Constraint (L1 Regularization)

Considering the persistent minute background vibrations during flat road driving, we introduce an L1 regularization term to construct a noise suppression mechanism:

(6) $$L_{Sparsity}=\|\hat{y}{\|}_{1}$$

This term applies sparsity induction during optimization, forcing the model to constrain predictive outputs to the physical noise floor level during non-shock intervals. This effectively mitigates the neural network’s overfitting and amplification of minor sensor noise, ensuring the controller maintains a low-response state under flat road conditions.

- Consistency Constraint

Relying solely on the L1 norm may cause the model to over-suppress valid minor shocks, leading to underfitting. To address this, we design a constraint mechanism based on adaptive spectral consistency. Utilizing the one-sided characteristic of ReLU, this mechanism establishes an energy lower bound based on the instantaneous input spectral energy ($E_{\mathrm{input}}$):

(7) $$L_{\mathrm{Consistency}}=\|\mathrm{ReLU}(\gamma E_{\mathrm{input}}^{\left(k\right)}-{\hat{y}}^{\left(k\right)}){\|}_{2}$$

where $E_{\mathrm{input}}^{\left(k\right)}$ denotes the aggregated time–frequency energy of the input signal for the *k*-th channel, and γ is a transmission coefficient (typically 0.5~1.0) accounting for system damping. This range is informed by established suspension isolation theory [37] and experimental observations [38] within the 0–20 Hz spectrum. This formula enforces an asymmetric causal constraint: When the physical input exhibits high energy, but the predicted value falls significantly below this excitation level, this term generates a strong gradient. This enforces the physical causality of “large excitation, necessary response”, ensuring the predicted energy does not breach the physical lower bound. When the prediction exceeds this lower bound, the loss term automatically zeroes out. At this point, the task of suppressing prediction amplitude is entirely dominated by $L_{\mathrm{Sparsity}}$.

Through this decoupled design of noise floor alignment (sparsity) and lower-bound protection (consistency), the algorithm effectively balances the conflict between false trigger rates and response sensitivity in semi-active control.

### 2.5. Design for Lightweight Deployment

Although CWT is employed for time–frequency analysis, the model architecture was designed from the outset with real-time deployment requirements on Electronic Control Units in mind:

- Parallel Preprocessing: Unlike RNN/LSTM architectures, which rely on historical states, the “Buffer-and-Crop” CWT strategy in this framework is fully parallelizable, allowing for efficient execution on GPUs or FPGAs.
- Shallow Convolutional Architecture: The PI-GCNN contains only three convolutional layers and replaces parameter-heavy fully connected layers with a GAP layer, significantly reducing computational complexity and memory footprint.

This lightweight design ensures inference completion within a millisecond-level control cycle (1–2 ms), providing theoretical feasibility assurance for practical engineering applications (specific inference performance tests are detailed in Section 4.3).

## 3. Simulation Experiments

This section establishes a high-fidelity virtual experimental environment based on the TruckSim (Version 2019.0) simulation platform, designed to evaluate the generalization performance and dynamic characteristics of the proposed PI-GCNN framework. The experimental procedure comprises two stages: first, constructing a foundational dataset containing typical road features for model training; and subsequently, conducting testing and validation on a specially customized track featuring combined driving conditions.

### 3.1. Simulation Environment Setup

#### 3.1.1. Vehicle Model

The 2A LCF Van (Two-Axle Low Cab Forward Van) from the TruckSim (Version 2019.0) library was selected to provide high-fidelity simulation of heavy commercial vehicle dynamics. Key configuration parameters include a 4 × 2 rear-wheel-drive layout, a wheelbase of 5000 mm, a total vehicle mass of approximately 5500 kg, and a center of mass height of approximately 1.18 m. The system simulation sampling frequency is set to 500 Hz.

#### 3.1.2. Dataset Generation

To construct a representative training dataset, we generated simulation data covering the following three categories of fundamental road features:

- Random Roads: ISO 8608 Class C and Class D road profiles were generated to simulate broadband random vibration excitations during driving [39].
- Symmetric Bumps: Simulating conditions where dual wheels traverse standard speed bumps synchronously, with obstacle heights distributed in the range of 30~60 mm
- Single-Sided Bumps: Configuring only single-sided wheels (left or right) to traverse obstacles to introduce asymmetric excitation, inducing vehicle roll motion.

Data collection totaled 96 groups, covering a wide speed range of 30~100 km/h.

#### 3.1.3. Data Balancing Strategy

The raw driving data exhibits a significant long-tail distribution: steady-state driving segments occupy the vast majority of time, whereas high-value shock condition samples are relatively sparse. Direct use of such raw data would likely cause the model to fall into local minima, rendering it insensitive to shock events. Consequently, we implemented the following data balancing strategy during the preprocessing stage:

- Transient events: Fully retaining all data segments containing significant road excitations.
- Steady-state segments: Performing down-sampling on flat road driving segments, retaining 10% of the original volume.

Post-processing, the final constructed dataset contains approximately $1.5\times 10^{5}$ samples, divided into training and validation sets at an 8:2 ratio.

#### 3.1.4. Implementation and Training Configuration

To ensure experimental reproducibility, the PI-GCNN model is built based on the PyTorch (Version 2.0.1) deep learning framework. All training and subsequent ablation studies were completed on a high-performance mobile computing workstation.

- Hardware Environment: Processor: Intel x86_64 architecture; Accelerator: NVIDIA GeForce RTX 4070 Laptop GPU.
- Software Environment: Python 3.9.23, PyTorch 2.0.1, CUDA 11.7.
- Training Hyperparameters: The model was trained for 100 epochs using the Adam optimizer with a batch size of 64. The initial learning rate was $1\times 10^{-3}$, decaying to a minimum of $1\times 10^{-6}$ via a Cosine Annealing scheduler. To balance the hybrid objective, the weights for the physics-guided loss ($\lambda_{\mathrm{phy}}$) and sparsity regularization ($\lambda_{\mathrm{sparse}}$) were set to 0.1 and 0.05, respectively. Additionally, the transmission coefficient *γ* in the physics-guided loss term was configured at 0.6.

#### 3.1.5. Evaluation Protocols: Quantitative and Qualitative

Given that a single numerical metric is difficult to fully reflect dynamic details (such as amplitude accuracy and response timeliness) when dealing with transient shocks, this study adopts a comprehensive evaluation strategy combining “Quantitative Metrics + Qualitative Visuals”.

1. Quantitative Metric: Pearson Correlation Coefficient

This metric primarily assesses the trend consistency between the predicted energy sequence and the physical ground truth response. For the performance of each dynamic mode *m* in a specific frequency band *b*, the Pearson Correlation Coefficient *R* between the prediction sequence $\hat{y}$ and the ground truth sequence y is calculated as:

(8) $$R_{m,b}=\frac{\sum_{i=1}^{N}({\hat{y}}_{i}-\bar{\hat{y}})(y_{i}-\bar{y})}{\sqrt{\sum_{i=1}^{N}({\hat{y}}_{i}-\bar{\hat{y}}{)}^{2}}\sqrt{\sum_{i=1}^{N}(y_{i}-\bar{y}{)}^{2}}}$$

where *N* is the total number of samples in the test set.

- Metric significance: Values of *R* closer to 1 indicate a better match in waveform morphology between the prediction and ground truth curves, implying the model accurately captures dynamic energy fluctuations.
- Frequency-band breakdown: We calculate *R* values for 12 channels (3 modes × 4 bands) individually to prevent low-frequency, high-energy signals from masking potential prediction deviations in high-frequency bands.

2. Qualitative Evaluation: Visual Inspection

Time-series comparison plots are introduced for intuitive verification. By superimposing the model prediction curve and the physical ground truth curve on the same time axis, we focus on examining two physical features:

- Peak fidelity: Checking the degree of overlap between the prediction and ground truth curves at the moment of shock occurrence to validate the model’s amplitude restoration capability and fitting precision for complex waveforms.
- Silent baseline: Verifying whether predicted values stably revert to minimal noise floor levels on flat road segments without shocks, thereby confirming the effectiveness of the gating mechanism in suppressing background noise.

#### 3.1.6. Test Track Configuration

To systematically evaluate the model’s generalization ability under non-training conditions, we constructed two distinct test tracks and defined expanded operating boundaries.

1. Track A (Off-road Severe-Mixed Scenario):

This track aims to simulate harsh, unpaved, or damaged road environments. It uses an ISO Class D random road as a base (introducing strong background high-frequency vibration), overlaid with dense asymmetric topological features:

- Staggered obstacles: Setting left and right wheels to traverse convex obstacles with different time delays, aiming to excite severe vehicle body torsion and roll motions.
- Single-sided potholes: Causing only one side’s wheels to fall into deep pits to test the model’s decoupled prediction capability under independent channel excitation.

2. Track B (Proving Ground: Bilateral Extreme):

This track simulates a standard proving ground environment without random background noise, containing a series of bilateral synchronous extreme excitation obstacles:

- High-speed bumps: Bilateral synchronous steps with a height of +15 cm.
- Deep potholes: Bilateral synchronous deep pits with a depth of −15 cm.

3. Expanded Boundary Conditions:

Beyond the unseen road profiles of Track A and Track B, the testing phase was evaluated under expanded operational boundaries. Unlike the typical operating conditions used for training (standard speeds of 30–100 km/h and normal payloads), the testing scenarios introduced extreme speeds (15–120 km/h) and a broader mass range spanning from partial load (3000 kg) to severe overload (9000 kg). These boundary configurations were excluded from the training set to verify the model’s out-of-distribution robustness.

### 3.2. Comparative Analysis with Baseline Methods

To validate the superiority of PI-GCNN in multi-modal energy prediction tasks, this section systematically compares it against two generic time-series prediction baseline models.

#### 3.2.1. Baseline Settings

To ensure the fairness of the comparative experiments, all baseline models adhere to a unified input-output configuration consistent with the PI-GCNN: the input consists of a historical sensor sequence of length $T_{total}=0.14\;s$ (including buffering), and the output corresponds to the modal energy evolution over the future $T_{pred}=0.5\;s$. The specific baseline models are as follows:

- 1D-CNN (Time-Domain Baseline): This model represents an end-to-end time-domain learning paradigm. It directly processes raw sensor signal sequences ($X\in {\mathbb{R}}^{3\times T}$), extracting temporal features through a 5-layer 1D convolutional network without involving explicit time–frequency transformation.
- Bi-LSTM (Sequential Baseline): Utilizes a Bidirectional Long Short-Term Memory network to process time-series signals. Given the LSTM’s advantage in capturing long- and short-term dependencies, this model serves as a standard baseline to evaluate the feature capture capability of Recurrent Neural Network (RNN) architectures under short observation windows.

#### 3.2.2. Quantitative Evaluation: Frequency-Domain Breakdown

To rigorously assess the generalization robustness of the PI-GCNN, this section adopts a cross-condition averaging strategy based on the OOD configurations defined in Section 3.1.6. The evaluation metrics presented in Table 1 and Table 2 represent the aggregate performance (measured by Pearson Correlation Coefficients) across the unseen combinations of Track A and Track B under the expanded operational boundaries. By evaluating the model on these zero-shot testing scenarios, we aim to verify its capability to map underlying vehicle dynamics rather than overfitting to specific training distributions.

1. Performance on Track A

Track A simulates an asymmetric excitation environment against a random road background. The average performance of each model across different frequency bands is detailed in Table 1.

Results Analysis: As indicated by the data in Table 1, the PI-GCNN (Ours) achieved superior prediction correlation across all 12 subdivided frequency bands. It is evident that as the frequency increases (from 0–3 Hz to 15–20 Hz), the performance of baseline models (particularly the 1D-CNN) exhibits significant degradation. For instance, in the Heave mode, the correlation coefficient of the 1D-CNN decreased significantly from 0.75 in the low-frequency band to 0.50. This suggests that purely time-domain convolution struggles to extract stable texture features from high-frequency discrete sampling points.

In contrast, the PI-GCNN maintains robust performance in high-frequency bands. This robustness is primarily attributed to the introduction of CWT time–frequency input, which converts high-frequency vibrations into texture patterns within scalograms. This 2D representation inherently possesses stronger noise immunity and feature stationarity compared to raw time-domain waveforms, thereby endowing the model with superior high-frequency generalization capabilities.

2. Performance on Track B

Track B features bilateral deep potholes (−15 cm) and high-speed bumps. The results are presented in Table 2.

Results Analysis: While all models exhibited suboptimal performance (R<0.6) in the low-frequency band (0–3 Hz) of the Heave mode—which involves severe vertical motion—the PI-GCNN demonstrated significant advantages in the Pitch and Roll modes. Taking the Pitch 0–3 Hz band as an instance, the correlation coefficient of Ours reached 0.85, substantially surpassing the 0.68 achieved by the 1D-CNN.

The core reason for this discrepancy lies in the preservation of phase structure. Extreme obstacles are often accompanied by complex wheel contact timing differences. Time-domain models tend to confuse this subtle temporal jitter with noise, whereas the CWT time–frequency map effectively preserves the phase structure of the signal. Consequently, even in the presence of deviations in vertical amplitude prediction, the PI-GCNN remains capable of accurately distinguishing whether the vehicle is in a pitching or rolling state, thereby guaranteeing high credibility in attitude prediction.

#### 3.2.3. Qualitative Visual Inspection

**Figure 4:**
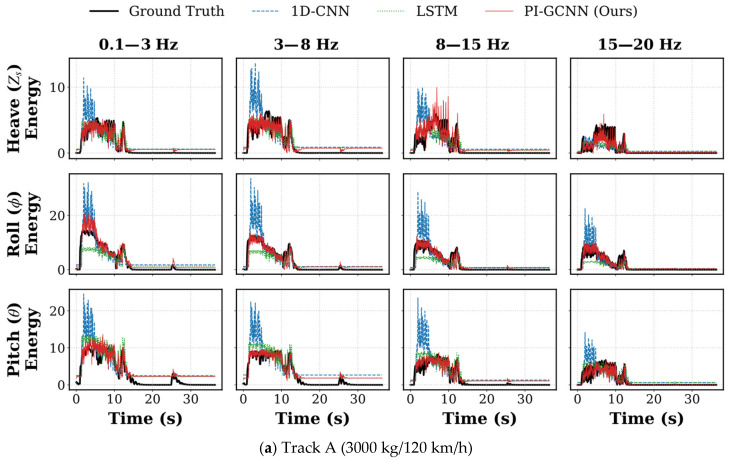

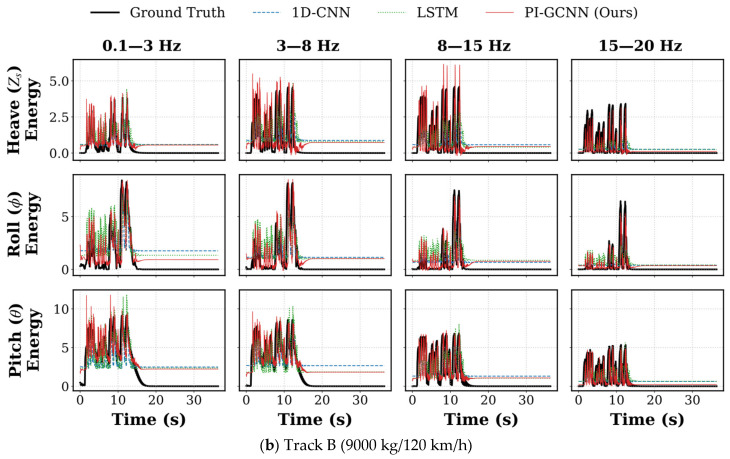
Detailed visualization of full-course energy prediction curves under varying operating scenarios. (**a**) Prediction performance on Track A (3000 kg/120 km/h); (**b**) Prediction performance on Track B (9000 kg/120 km/h). In each panel, the results are organized into a 3 × 4 grid, representing the heave, roll, and pitch dynamics across four distinct frequency bands. To facilitate direct comparison, the predictive curves of the proposed PI-GCNN (red solid lines), 1D-CNN (blue dashed lines), and LSTM (green dotted lines) are overlaid against the ground truth (thick solid black lines).

To visually analyze the behavioral differences underlying the quantitative metrics, we selected full-course prediction data from two typical scenarios—Track A (3000 kg/120 km/h) and Track B (9000 kg/120 km/h)—for comparison (see Figure 4). By observing the fitting details between the prediction curve (red line) and the ground truth (black line), it is evident that baseline models exhibit distinct structural biases when processing non-stationary signals.

1D-CNN: As shown by the blue line, the 1D-CNN exhibits excessive sensitivity to local high-frequency features, leading to divergence at amplitude extremes. When encountering strong excitations (such as the deep potholes in Track B), the model tends to amplify local texture features, producing significant overshoot, where predicted peaks are far higher than the true energy. Conversely, in low-energy decay regions, the model struggles to suppress background noise, demonstrating steady-state deviation. This indicates that purely time-domain convolution finds it difficult to maintain gain stability across a wide dynamic range.

LSTM: The LSTM prediction trajectory (green line) presents a strong tendency toward mean regression. Limited by the memory update mechanism of recurrent units, the model tends to output a weighted average of historical information, thereby exhibiting conservative peak attenuation characteristics. Particularly during millisecond-level energy transients under 120 km/h conditions, the LSTM fails to respond in a timely manner to large signal transitions, causing critical shock peaks to be smoothed out. This systematic underestimation may cause the semi-active control system to suffer from insufficient damping force when it is most needed.

PI-GCNN: In contrast, the PI-GCNN (red line) demonstrates superior balance across the full dynamic range. Benefiting from the effective separation of transient structures and steady-state textures by CWT features, the model avoids the 1D-CNN’s over-reaction to noise while overcoming the LSTM’s smoothing bias. Whether in the rising edge of extreme shocks or the oscillation zones of random roads, the PI-GCNN achieves tight tracking of energy amplitude, proving its robustness in processing non-stationary vehicle dynamics signals.

### 3.3. Ablation Study

To verify the effectiveness and necessity of the core components within the PI-GCNN framework, we conducted ablation experiments on Track A and Track B.

#### 3.3.1. Ablation Variants

To decouple the contributions of different modules, we constructed the following three baseline variants. All variants uniformly employ Huber Loss as the fundamental regression loss. The definitions of each variant are as follows:

- M0 (Vanilla 2D-CNN): Uses only the CWT time–frequency map as input without any physical constraint mechanisms. This represents the baseline performance of a purely data-driven approach.
- M1 (+Physics Loss): Introduces sparse physics loss (incorporating the L1 sparsity term and the adaptive spectral consistency term) on top of M0, but removes the gating branch. This aims to evaluate the contribution of the physically constrained loss function.
- M2 (+Gating Branch): Introduces the physical gating branch on top of M0. This aims to evaluate the physical truncation capability of the gating structure itself.
- Ours (Full System): The complete PI-GCNN framework, containing both the physical gating branch and the physics loss function.

#### 3.3.2. Quantitative Comparison

Table 3 and Table 4 present the average Pearson Correlation Coefficients *R* of each variant across different frequency bands.

Results Analysis: A step-wise improvement in performance can be clearly observed from the tabulated data.

- Limitations of M0: M0 performed the worst across all frequency bands, particularly in the high-frequency band of Track B (Roll 15–20 Hz was only 0.34), indicating that purely data-driven models struggle to extract robust features from high-noise data.
- M1 vs. M2: The magnitude of performance improvement in M1 (introducing physics loss) was generally superior to that of M2 (introducing gating only). This suggests that without the guidance of an explicit optimization objective (loss), the gain yielded by solely augmenting the network structure (gating) is limited.
- Combined Advantage of Ours: The full system, which combines the advantages of both, achieved the best performance in the vast majority of frequency bands, thereby proving the existence of a positive coupling effect between the architecture and the loss function.

#### 3.3.3. Analysis of Component Effectiveness

**Figure 5:**
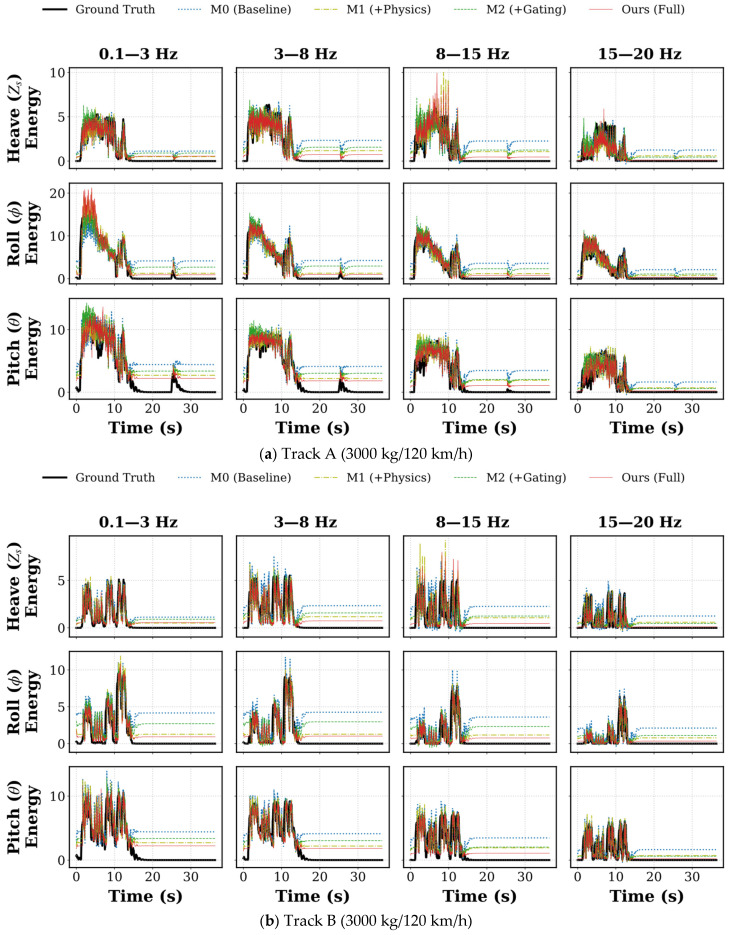
Detailed visualization of ablation study results comparing the proposed PI-GCNN with its ablated variants under different scenarios. (**a**) Comparison results on Track A (3000 kg/120 km/h); (**b**) Comparison results on Track B (3000 kg/120 km/h). Similar to the main results, each panel is structured in a 3 × 4 grid to display the three motion modes across four frequency bands. The performance curves of the proposed PI-GCNN (red solid lines), Model M0 (blue dotted lines), Model M1 (golden-yellow dash-dotted lines), and Model M2 (green dashed lines) are directly overlaid against the ground truth (thick solid black lines) to clearly demonstrate the progressive improvement in prediction fidelity.

To investigate the underlying mechanisms behind the quantitative improvements, we selected full-course energy prediction curves from two typical driving conditions for comparison (see Figure 5).

1. Baseline Regression Bias (M0): As shown by the blue line in Figure 5, the M0 model outputs the highest energy levels during flat road intervals, which are significantly higher than the ground truth. This regression bias stems from the absence of sparsity constraints. When processing long-tail data, standard regression loss functions typically exhibit L2 behavior in low-error regions, failing to drive the output to a hard zero. Consequently, the network’s bias parameters tend to be inflated by high-amplitude shock samples, resulting in a persistent baseline offset during non-impact intervals.
2. Gating without Sparsity (M2): Although M2 (green line) incorporates a gating branch, its output on flat roads is only slightly lower than that of M0, with significant numerical residuals persisting. This indicates that the gating structure alone is insufficient to eliminate the noise floor. In the absence of a sparsity penalty, the optimizer tends to keep the gating parameters in a “partially open” state to prevent underfitting at moments of shock. As a result, the gating coefficient *g* does not converge to extremely low values during flat segments, leading to incomplete truncation.
3. Effect of Regularization (M1): In contrast, the flat road output of M1 (orange line) is significantly reduced, lower than both M0 and M2, without exhibiting signal loss. This improvement results from the combined action of the L1 sparsity term and the spectral consistency term. The L1 term provides a constant gradient that forces the optimizer to compress network weights during flat segments, effectively reducing background noise amplitude. Meanwhile, the spectral consistency constraint acts as a physical lower bound; when the predicted value falls below the physical input energy, it generates a penalty gradient to boost the gain. This combination allows M1 to suppress the noise floor while maintaining responsiveness to weak signals.
4. Architecture-Objective Fit (Ours): The proposed PI-GCNN (red line) achieves the lowest output on flat roads, and its dynamic response aligns most closely with the ground truth. This is because the framework applies the optimization objective of M1 directly to the gating structure of M2, achieving efficient parameter optimization. Unlike M1, which relies on adjusting the convolutional weights of the backbone network to satisfy sparsity, the full model can quickly minimize the L1 loss by directly lowering the gating coefficient g. This alignment between the objective function and network structure enables the model to minimize numerical residuals at minimal cost, while simultaneously maintaining sensitivity to transient shocks through the spectral consistency constraint.

## 4. Experimental Verification

To validate the generalization capability and prediction timeliness of the proposed model under real-world conditions, this chapter conducts cross-condition testing based on the public PVS 9 (Probe Vehicle System) dataset. Collected via high-precision sensors, this dataset captures the suspension response characteristics of real vehicles on unstructured roads.

### 4.1. Dataset and Experimental Setup

The experiment utilized driving records of the Fiat Palio model from the dataset, specifically the session identified as the 310.74 km segment (Driver 3). This segment contains significant non-stationary road features (such as speed bumps, potholes, and irregular undulations), which effectively excite broadband vibrations of the vehicle across three degrees of freedom: Heave, Roll, and Pitch. It serves as an ideal scenario for testing the transient response capability of the prediction model.

In our validation, the heave acceleration, roll rate, and pitch rate were specifically extracted from the IMU mounted on the vehicle dashboard. For detailed visual photographs of the physical vehicle instrumentation and the full experimental setup, readers are referred to the original dataset publication [35].

In the data preprocessing stage, raw on-board accelerometer and gyroscope data were resampled to 500 Hz to match the model’s input sampling rate requirements. To evaluate the practical efficacy of the algorithm, we compared the proposed PI-GCNN with a real-time baseline. Here, the PI-GCNN outputs the modal energy evolution for the future 0.5 s, whereas the real-time baseline is based on CWT calculation results from the past 0.14 s, representing the timeliness level of traditional monitoring methods.

### 4.2. Analysis of Experimental Results

**Figure 6:**
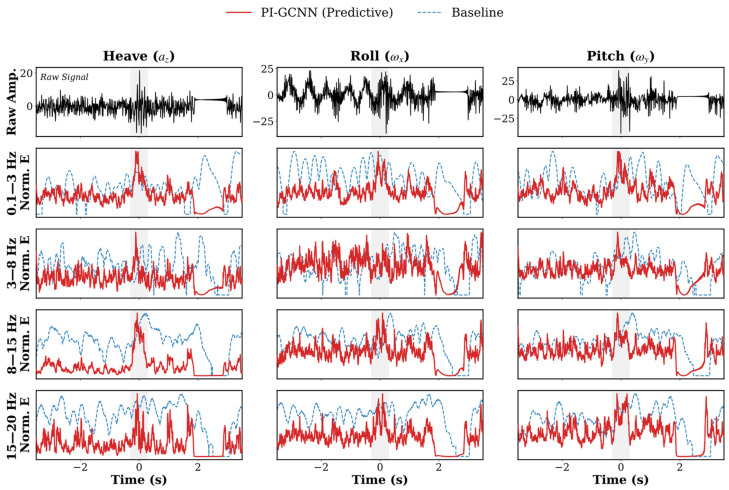
Experimental validation using real-vehicle data. The results are unified into a 5 × 3 grid, where the three columns correspond to the Heave acceleration, Roll rate, and Pitch rate, respectively. For each motion mode, the top row displays the raw time-domain signal (black line), with the gray shaded region marking the critical interval of the physical shock peak. The subsequent four rows compare the predicted energy (red solid lines) against the real-time baseline (blue dashed lines) across four distinct frequency bands. This comprehensive layout explicitly illustrates the predictive phase lead of the proposed model relative to the actual impact event.

Figure 6 presents the comparative results of energy prediction for three degrees of freedom across different frequency bands (0.1–20 Hz) when the vehicle traverses obstacles at approximately 30 km/h.

By comparing the predicted energy curve (red line), the raw time-domain signal (black line), and the real-time baseline (blue dashed line), the following key conclusions can be drawn:

1. Significant Phase Lead

Observing the Heave (3–8 Hz) band in and the Pitch (3–8 Hz, 8–15 Hz) bands in Figure 6, a significant temporal discrepancy is evident:

- Physical Response Lag: The raw time-domain signal (black line) exhibits large oscillations only around *t* = 0 s, marking the physical moment when wheel-obstacle contact reaches peak vibration.
- Predictive Response Lead: The predicted energy of the proposed model (red line) begins to climb and peaks within the interval of *t* = −0.2 s to *t* = −0.1 s.

This result indicates that the model successfully extracted precursor features prior to the shock from the CWT time–frequency maps. This characteristic reserves an actuation window of approximately 100–200 ms for the active suspension system, enabling it to adjust damping force in advance to cope with the impending shock.

2. Overcoming Inherent Lag of Real-time Methods

In contrast, because the real-time baseline (blue dashed line) relies on the processing of historical observation, its energy peak inevitably appears after the physical shock occurs (t>0 s). This causal lag is a primary source of time delay in traditional semi-active suspension control. By transforming the monitoring task into a sequence prediction task, PI-GCNN effectively mitigates this physical limitation, realizing a shift from reactive control to feedforward control.

3. Noise Suppression in Non-Impact Intervals

On flat road segments before and after shocks, the model exhibits distinct noise suppression characteristics. As shown in Figure 6, the curve of the real-time baseline method still contains persistent numerical fluctuations during non-shock intervals, failing to completely filter high-frequency inputs caused by road texture. Conversely, benefiting from the truncation effect of the gating mechanism, the predicted energy of PI-GCNN decays rapidly after passing obstacles and stabilizes at the noise floor level. This characteristic of low numerical residuals under steady-state conditions helps reduce false actions in the control system and avoids unnecessary actuator intervention.

### 4.3. Computational Efficiency and Deployment Feasibility

To validate the deployment potential of the algorithm in engineering applications, we evaluated the computational complexity and inference latency of the PI-GCNN model.

- Model Complexity: Benefiting from the compact 3-layer convolutional design, the model’s total parameters are only 0.10 M, and the weight file storage occupies less than 1 MB. This lightweight architecture avoids reliance on computationally intensive operators (such as large-scale Transformer attention matrices), making it suitable for operation on resource-constrained edge devices.
- Inference Latency: On the experimental platform (NVIDIA RTX 4070 Laptop), the average latency for processing a single frame is 0.25 ms. This consists of 0.03 ms for CWT preprocessing and 0.22 ms for CNN inference, corresponding to a throughput of approximately 4000 FPS.
- Deployment Feasibility: Typical control cycle requirements for active suspension systems range from 10~20 ms. Even if the model is migrated to low-power embedded platforms (such as NVIDIA Jetson Nano or automotive-grade MCUs) with only 1/50~1/100 the computing power of the experimental platform, the estimated theoretical inference latency remains controllable within the 10–20 ms range. This indicates that the algorithm possesses sufficient Computational Margin to meet Real-Time Control requirements on automotive-grade chips.

## 5. Conclusions

This paper proposes a Physics-Informed Gated Convolutional Neural Network (PI-GCNN) framework to address the inherent conflict between response latency and noise disturbance in semi-active suspension control. By integrating the Continuous Wavelet Transform (CWT) for time–frequency feature extraction with a physics-guided gating mechanism, the method achieves accurate multi-modal energy prediction under complex dynamic conditions. Combining high-fidelity TruckSim (Version 2019.0) simulations and validation on the public PVS 9 real-vehicle dataset, the main conclusions are drawn as follows:

1. Robust Generalization across Conditions: Simulation experiments confirmed that the PI-GCNN accurately captures non-linear vehicle dynamic responses. Across a wide range of operating conditions—including varying loads (3000–9000 kg), speeds (15–120 km/h), and heterogeneous obstacles (bumps/potholes)—the predicted energy envelopes maintained high consistency with the physical ground truth. This indicates that the model successfully learned the underlying physical mapping of vehicle dynamics rather than merely fitting training data.
2. Superior Control Performance and Noise Robustness: In real-vehicle validation, the model successfully extracted transient precursors from initial road impacts, achieving a significant phase lead before the high-energy modal vibrations fully developed. This anticipatory capability provides a critical actuation window to compensate for the inherent mechanical delays of semi-active actuators. Simultaneously, the physics-guided gating mechanism effectively suppressed background noise during steady driving, ensuring the robustness of the feedforward perception.
3. Feasibility for Embedded Deployment: Benefiting from the compact convolutional architecture, the model contains only 0.10 M parameters. The single-frame inference latency on a mobile computing platform is approximately 0.25 ms, which is significantly lower than the typical 10–20 ms control cycle of active suspension systems. This demonstrates sufficient computational margin for real-time deployment on resource-constrained automotive-grade embedded chips.

Future Work: Subsequent research will focus on two directions: (1) integrating the predicted energy matrix into a closed-loop control strategy to quantitatively evaluate the improvement in whole-vehicle ride comfort and handling stability; and (2) introducing road adhesion coefficient estimation to investigate the impact of variable friction conditions on dynamic responses, thereby expanding the model’s applicability under complex weather environments.

## Figures and Tables

**Table 1:** Detailed Pearson Correlation *R* on Track A.

Mode	Heave				Pitch				Roll			
Freq Band	0–3 Hz	3–8 Hz	8–15 Hz	15–20 Hz	0–3 Hz	3–8 Hz	8–15 Hz	15–20 Hz	0–3 Hz	3–8 Hz	8–15 Hz	15–20 Hz
LSTM	0.7216	0.6450	0.5760	0.5212	0.8237	0.8048	0.7717	0.7349	0.8993	0.8901	0.8712	0.8685
1D-CNN	0.7461	0.5863	0.5049	0.5790	0.8092	0.7757	0.7197	0.7234	0.8562	0.8435	0.8101	0.7966
Ours	0.8726	0.7816	0.7741	0.7917	0.9099	0.9082	0.9020	0.9268	0.9304	0.9284	0.9233	0.9268

**Table 2:** Detailed Pearson Correlation *R* on Track B.

Mode	Heave				Pitch				Roll			
Freq Band	0–3 Hz	3–8 Hz	8–15 Hz	15–20 Hz	0–3 Hz	3–8 Hz	8–15 Hz	15–20 Hz	0–3 Hz	3–8 Hz	8–15 Hz	15–20 Hz
LSTM	0.7809	0.5968	0.4771	0.4595	0.7612	0.7786	0.7807	0.7956	0.6961	0.6855	0.5961	0.5908
1D-CNN	0.7575	0.5313	0.4348	0.4720	0.6805	0.6646	0.6965	0.7205	0.7104	0.7096	0.6945	0.7274
Ours	0.7942	0.5907	0.4825	0.5472	0.8500	0.8548	0.7941	0.8360	0.8306	0.7746	0.7555	0.8175

**Table 3:** Ablation Results on Track A.

Mode	Heave				Pitch				Roll			
Freq Band	0–3 Hz	3–8 Hz	8–15 Hz	15–20 Hz	0–3 Hz	3–8 Hz	8–15 Hz	15–20 Hz	0–3 Hz	3–8 Hz	8–15 Hz	15–20 Hz
M0 (Baseline)	0.8532	0.6962	0.6276	0.6435	0.8721	0.8663	0.8013	0.8609	0.9033	0.8623	0.8467	0.8804
M1 (+Physics)	0.8616	0.7609	0.7441	0.7463	0.9013	0.9028	0.8790	0.9026	0.9269	0.9244	0.9153	0.9152
M2 (+Gating)	0.8695	0.7728	0.7369	0.7639	0.8917	0.8989	0.8914	0.9183	0.9287	0.9025	0.8936	0.9180
Ours (Full)	0.8726	0.7816	0.7741	0.7917	0.9099	0.9082	0.9020	0.9268	0.9304	0.9284	0.9233	0.9268

**Table 4:** Ablation Results on Track B.

Mode	Heave				Pitch				Roll			
Freq Band	0–3 Hz	3–8 Hz	8–15 Hz	15–20 Hz	0–3 Hz	3–8 Hz	8–15 Hz	15–20 Hz	0–3 Hz	3–8 Hz	8–15 Hz	15–20 Hz
M0 (Baseline)	0.7290	0.4130	0.2825	0.3420	0.7630	0.7284	0.5933	0.7054	0.6223	0.4996	0.4902	0.6172
M1 (+Physics)	0.7994	0.5462	0.4218	0.4884	0.8326	0.8357	0.7442	0.8037	0.8135	0.7612	0.7333	0.7806
M2 (+Gating)	0.7724	0.5110	0.4136	0.5423	0.8164	0.8049	0.7454	0.8201	0.7453	0.5973	0.5822	0.7292
Ours (Full)	0.7942	0.5907	0.4825	0.5472	0.8500	0.8548	0.7941	0.8360	0.8306	0.7746	0.7555	0.8175

## Data Availability

The PVS 9 dataset analyzed in this study is publicly available at [https://www.kaggle.com/datasets/jefmenegazzo/pvs-passive-vehicular-sensors-datasets (accessed on 4 March 2026)]. The simulation data generated during the study are available from the corresponding author upon reasonable request.
